# *The SNPcurator*: literature mining of enriched SNP-disease associations

**DOI:** 10.1093/database/bay020

**Published:** 2018-03-08

**Authors:** Noha S Tawfik, Marco R Spruit

**Affiliations:** 1 Computer Engineering Department, College of Engineering, Arab Academy for Science, Technology, and Maritime Transport (AAST), Abukir,1029 Alexandria, Egypt; 2 Department of Information and Computing Sciences, Utrecht University, Princetonplein 5, 3584 CC Utrecht, The Netherlands

## Abstract

The uniqueness of each human genetic structure motivated the shift from the current practice of medicine to a more tailored one. This personalized medicine revolution would not be possible today without the genetics data collected from genome-wide association studies (GWASs) that investigate the relation between different phenotypic traits and single-nucleotide polymorphisms (SNPs). The huge increase in the literature publication space imposes a challenge on the conventional manual curation process which is becoming more and more expensive. This research aims at automatically extracting SNP associations of any given disease and its reported statistical significance (*P*-value) and odd ratio as well as cohort information such as size and ethnicity. Our evaluation illustrates that SNPcurator was able to replicate a large number of SNP-disease associations that were also reported in the NHGRI-EBI Catalog of published GWASs. SNPcurator was also tested by eight external genetics experts, who queried the system to examine diseases of their choice, and was found to be efficient and satisfactory. We conclude that the text-mining-based system has a great potential for helping researchers and scientists, especially in their preliminary genetics research. SNPcurator is publicly available at http://snpcurator.science.uu.nl/.

**Database URL**: http://snpcurator.science.uu.nl/

## Introduction

Ever since its completion in 2003, the Human Genome Project has accelerated and encouraged research on decoding the genome structure and functionality, powered by the huge advances in the genotyping technologies. The main goal of genomic studies is to identify and reveal the genetic variations associated with diseases and its prevalence across different populations. Such studies contribute to more tailored detection, prevention and treatment of diseases which lay the groundwork for the era of personalized medicine (1). In the hunt for correlation between genotype and phenotype, single-nucleotide polymorphisms (SNPs) are considered genetic signatures to the majority of polymorphisms responsible for trait susceptibility (2).

By definition, a SNP is a single base-pair (A, T, C or G) variation that occurs at a specific site in the DNA sequence. It does not directly cause a disease but increases the genetic predisposition of individuals towards a certain disease and can affect their responses to drugs and medications (3). Currently, information on SNPs is available in databases such as the genome-wide association study (GWAS) Catalog (4), Gwas Central (5), GWASdb (6), mirsSNP (7), GRASP (8). These resources are constructed and curated manually; however, and the richness of information specifically related to the clinical impact of SNPs is contained in free text in the form of biomedical publications (9). The process of updating databases requires substantial human resources, financial support not to mention time (4). This imposes a challenge as the number of published studies is steadily increasing and hence the manual curation is proving more and more inefficient.

Text-mining tools have been employed recently to overcome the mentioned limitations and accelerate the curation process. Mutation finder (MF) extracts mutations through regular expressions while tmVar (10) also extracts mutations based on conditional random fields. Open Mutation Miner (OMM) (11) uses MF to recognize single mutations and extends its regular expression set to detect mutation series. The extractor of mutations (EMUs) (12) detects mutations in text and links them to genes, proteins and diseases. The SNP Extraction Tool for Human Variations (SETH) (13) implements an Extended Backus–Naur Form and regular expressions with more emphasis on short sequence variations and SNPs. Disgent database (14) lists results compiled from expert curated databases and enhances the results by incorporating the BeFree text-mining system (15). Polysearch (16) associates genetic variants to diseases and drugs based on their co-occurrence frequency in abstracts.

Most of the above tools achieved high performance levels on different corpora. However, there is still a gap between the research community and the biomedical text-mining community. Yepes and Verspour compare in (17) the performance of EMU, OMM, MF, tmVar and SETH intrinsically on the Variome corpus, and extrinsically on the COSMIC and InSiGHT database. The study also discusses the technical aspects related to using the tools; some of them require an intermediate to advanced level of programming knowledge to use them. Furthermore, the evaluation of the practical utility of the tools is not properly investigated nor how can they be adapted to fulfill a researcher’s tasks efficiently.

In this article, we present the SNPcurator, a system more oriented towards information extraction specifically in the genome wide and candidate genes studies. The proposed model is constructed out of different natural language processing (NLP) modules to aid scientists in their search for relevant disease-associated SNPs through an intuitive web interface. It incorporates both syntactic and semantic methods to extract relevant information from PubMed abstracts such as cohort size and ethnicity, SNP ids and the reported results. The motivation behind this research is to create a publically available, scalable and fully automated extraction tool.

## Materials and methods

**Figure 1.**
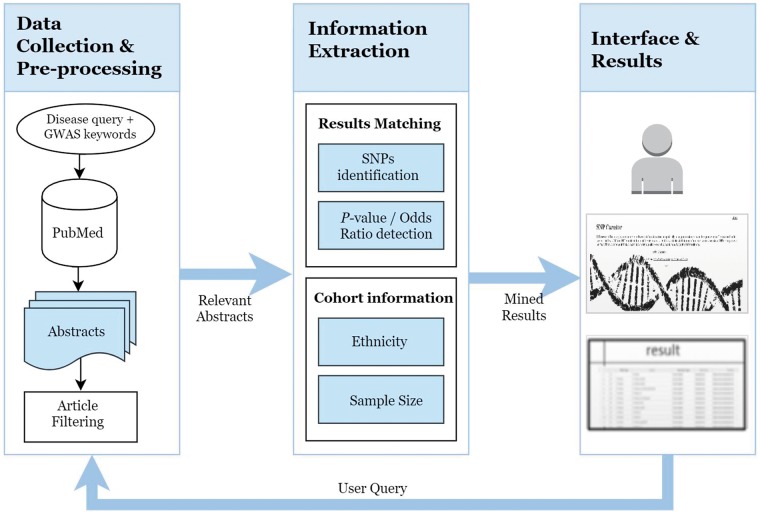
The SNPcurator workflow.

SNPcurator has an online web interface at http://snpcurator.science.uu.nl/ and allows researchers to easily query and search diseases and provides a useful resource for overview and summarization of associated SNPs found in literature. Sample code and the files used for evaluation are also available for download. Figure 1 illustrates the system’s overview and workflow.

## Data collection

The first step is to identify genetic research studies from the complete PubMed repository without any limitation on citations counts, publishing journals or a certain time period. The NCBI Eutilies (https://eutils.ncbi.nlm.nih.gov/entrez/eutils/), in particular Esearch and Efetch, are used in conjunction with the BioPython module to query the PubMed database. All articles included in PubMed are associated with Medical Subject Headings (MeSH) terms (http://www.ncbi.nlm.nih.gov/mesh). MeSH is a controlled vocabulary used for indexing articles according to the unified Medical Language System meta-thesaurus which powers up the search capability of PubMed. SNPcurator combines the user input (the disease term in the current version) with relevant genetic search terms to construct the following PubMed query:

‘{disease} AND(“Polymorphism, Single Nucleotide”[Mesh Terms] OR “Genetic Predisposition to Disease”[Mesh Terms] OR “Genome-Wide Association Study”[Mesh Terms])’

*Esearch* returns a list of IDs that matches the query search while *Efetch* returns data records for the retrieved ID list. Not all retrieved articles are included in the final search results; as the system only relies on the information found in abstracts and not the full-text article, all records with missing/incomplete abstract text or in a foreign language are excluded. The downloaded results will include literature with research on multiple genetic variants such as mutations or genes. To limit our search scope to SNP-association studies only, we apply a second filter to determine if the abstract text is relevant by checking SNP occurrences in text.

## Information extraction

The filtered results are then pre-processed by several NLP modules. SNPcurator employs the publically available spaCy toolkit which is optimized for both accuracy and performance (https://spacy.io/). It accomplishes all necessary NLP tasks easily through its Python API. SpaCy is debatably one of the fastest publicly available parsers (18). We make use of its sentence splitting, tagging, tokenization, name entity recognition and dependency parsing modules to extract SNP-disease pairs.

### SNPs identification

Scientists refer to SNPs in their research through unique ID numbers in a standard format assigned by the NCBI dbSNP database (http://www.ncbi.nlm.nih.gov/SNP/). The ID format differs between a newly submitted SNP (e.g.: *ss28937569)* or reference SNP cluster, refSNP (e.g.: *rs28937569*), where the latter is assigned to a submitted SNP after the sequence is aligned to its appropriate region (19). A regular expression formula ‘[rR][sS] []*[0-9][0-9]*’ is used to identify the SNP identifier format and extract a list of SNP occurrences in the abstract text. This expression has previously achieved a 100% recall over a test set of 300 SNP mentions (20). In our model, the latter formula has been improved to optionally accept characters *G, C, T* and *A* at the end of SNP mentions as authors tend to specify reference/alternative alleles or chromosome position in a non-standard format.

### Results matching

In the analysis of genetic variations, researchers follow exhaustive guidelines and conduct several statistical tests in order to report positive associations in GWAS which in return requires the definition of a known threshold. *P-*value, a parameter of statistical significance is used to determine the certainty of an association. It provides the probability that a given result from a test is due to chance with lower *P-*values giving higher probability of the association. Many values have been reported as a threshold to attain genome-wide significance (21). Therefore, our model includes all studies even those with marginally significant and insignificant *P*-values. By displaying all results without filtering their significance, SNPcurator aims to help researchers rule out a given hypothesis or trigger more questions for further investigations.

To relate a reported value(s) to mentioned SNPs, the system first highlights one or more ‘result sentence(s)’ from the abstract. A ‘result sentence’ is identified as any phrase with *P*-value mention along with a numeric value. Each flagged sentence is first tokenized into words and then pre-processed to remove any unnecessary characters like quotes and brackets. There are also a number of naming patterns for reporting a *P*-value according to how it was calculated (e.g. ‘*P*-combine’, ‘*P*-value’, ‘*P*-meta’), which requires normalization to one single standard format that is easy to capture and extract. Following that, a set of predefined regular expressions are used to capture and recognize the format followed by floating, scientific or exponent number notations. However, since a sentence meaning relies on its semantics and structure, the coupling of *P*-value to its corresponding SNP is not the same for all sentences. The most common and straight forward way of stating results is by mentioning both SNP and *P*-value in the same sentence. In this type, the extraction of needed pairs <SNP, *P*-value> relies on the order as illustrated in the examples shown in Table 1. The system forms a pair by extracting the nearest *P*-value to the SNP mentions. Note that a detected *P*-value can only be coupled with a single SNP mention, and thus not considered when measuring the distance for the next SNP. This allows an accurate coupling when multiple values are involved such as in examples (2, 3 and 4). The same process also applies to the extraction of the odds ratio (OR) values reported in abstract text. In general, OR is a measure of the effect size in case-control study and in genomic-studies specifically, it denotes the probability of having the disease n individuals with and without certain genotypes of SNPs.

**Table 1.** Examples of (SNP, P/OR values) pairs extracted from evidence sentences

PubMed ID (PMID)		Evidence sentence
1	21552555	We next examined obesity-related quantitative traits such as total body weight, waist circumference and waist to hip ratio, and detected genome-wide significant signals between waist to hip ratio and NRXN3 rs11624704,*P* = 2.67 ×10^−9^, previously associated with body weight and fat distribution.
2	23143601	We identified three new susceptibility loci at 10q25.2 (rs7086803, *P* = 3.54 × 10(−18)), 6q22.2 (rs9387478, *P* = 4.14 × 10(−10)) and 6p21.32 (rs2395185, *P* = 9.51 × 10(−9)).
3	24880342	We identified large-effect GWASs for squamous lung cancer with the rare variants BRCA2 p.Lys3326X (rs11571833, OR = 2.47, *P* = 4.74 × 10(−20)) and CHEK2 p.Ile157Thr (rs17879961, OR = 0.38, *P* = 1.27 × 10(−13)).
4	21725308`	The combined analyses identified six well-replicated SNPs with independent effects and significant lung cancer associations (*P* ≤ 5.0 × 10(−8)) located in TP63 (rs4488809 at 3q28, *P* = 7.2 × 10(−26)), TERT-CLPTM1L (rs465498 and rs2736100 at 5p15.33, *P* = 1.2 × 10(−20) and *P* = 1.0 × 10(−27), respectively), MIPEP-TNFRSF19 (rs753955 at 13q12.12, *P* = 1.5 × 10(−12)) and MTMR3-HORMAD2-LIF (rs17728461 and rs36600 at 22q12.2, *P* = 1.1 × 10(−11) and *P* = 6.2 × 10(−13), respectively).

### Patient information extraction

The sample size included in the study is another key parameter when confirming the association with statistical confidence. For that purpose, we created two sets of keywords that are commonly used to describe both the patient cohort (‘PatientKeywordSet’) and the control group (‘ControlKeywordSet’). The ‘PatientKeywordSet’ consists of words such as [‘patient’, ‘case’, ‘subject’] and the ‘ControlKeywordSet’ includes words like [‘control’, ‘normal’, ‘healthy’]*.* The spaCy parser is then invoked to search for all numeric modifier (NUMMOD) dependencies found in the abstract text. The keyword sets are first compared against the head token of each candidate modifier and in the case of a no match; they are then compared against a list of two neighboring words of the candidate. Examples of control and patient group sizes extracted from evidence sentences are demonstrated in Table 2.

**Table 2.** Examples of control and patient group sizes extracted from evidence sentences

PubMed ID (PMID)		Evidence sentence
5	26141218	We genotyped IL1B SNPs in a case-control study with 889 lung cancer cases and 1005 controls using the SNPscan Genotyping system.
6	25245582	A total of 169 ED patients (106 with anorexia nervosa (AN) and 63 with bulimia nervosa (BN)) and 312 healthy subjects were genotyped.

Because genetic variants often have markedly different frequencies across populations, the system is also able to extract the ethnicity and nationality of patients through the spaCy name entity recognition module. The *ents* attribute of the processed input abstract lists all entity objects found. An entity object is a combination of text, category and word position within text. We are particularly interested in the NORP entity which extracts nationalities, religious or political groups efficiently (where the latter two are irrelevant in this context). To limit the number of false positives, we also set the minimum word length for a nationality. If more than one nationality/ethnicity are found, the most frequent nationality mentioned would be reported as shown in Table 3.

**Table 3.** Examples of nationalities and ethnicities extracted from evidence sentences

PubMed ID (PMID)		Evidence sentence
7	22399527	We conducted a GWA study on MetS and its component traits in 4 Finnish cohorts consisting of 2637 MetS cases and 7927 controls.
8	22399527	Therefore, we explored the association between the polymorphisms of CTSS and metabolic disorders in a Chinese Han population.

## Interface

**Figure 2.**
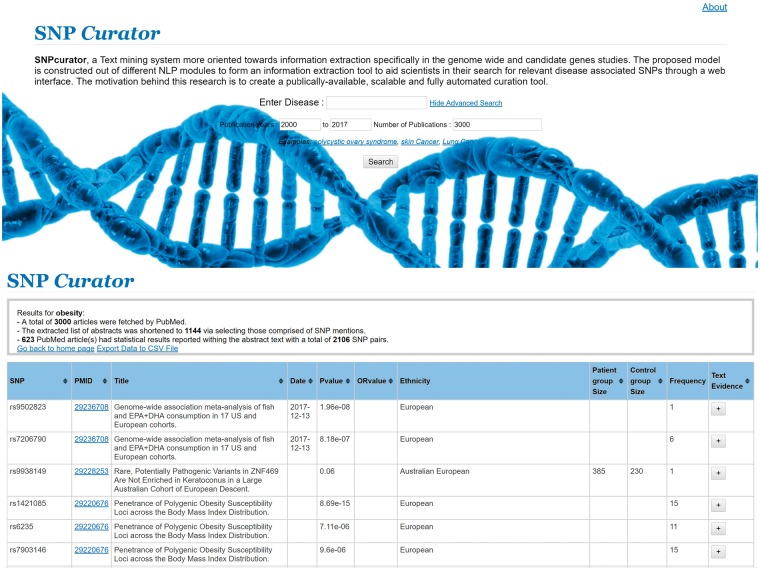
The SNPcurator web interface.

SNPcurator web service is implemented in Python and based on the Flask web development library (http://flask.pocoo.org/). Flask is a micro-framework for Python based on Werkzeug, the WSGI utility library and Jinja temple engine. It provides simple and flexible routing to build python-based web applications and also allows the integration of other python scripts. The disease query is passed from the front-end; the server initiates and displays the output from the text-mining module script. All the extracted information is displayed in a tabular format, where each row includes a single SNP item. The last column allows the user to check the result sentence from which the <SNP, *P*-value> tuple was extracted. The user is able to sort the results according to the *P*-value, OR value, group sizes, date of publication or simply alphabetical/numerical order of the PubMed or SNPs Ids. For example, by ranking results according to the ascending *P*-values, researchers can easily view the SNPs with the strongest association values that attained the genome-wide significance level. The web-tool is deployed on a server connected to the internet and can be accessed at http://snpcurator.science.uu.nl/, Figure 2 shows screenshots of SNPcurator’s interface.

## Evaluation and discussion

### Results

To assess SNPcurator’s performance, we compared the information extracted from SNPcurator to data found in the GWAS Catalog for two queries: Obesity and Lung Cancer. According to cancer.org, Lung cancer is the second most common type of cancer that affects both men and women (combined) and it is the first cause of cancer death. Although obesity is not in itself a direct cause of death, overweight is a major risk factor of several diseases leading to death. However, it is also considered one of the top preventable diseases. Therefore, by identifying individuals with increased risk of obesity, an early treatment plan would help to avoid mortal consequences (22).

SNPcurator was able to extract 1422 associations while the GWAS catalog shows a greater number of results with evidence of 1887 associations for the obesity trait. For the lung cancer disease, SNPcurator shows 620 associations versus 629 found in the GWAS catalog. This was expected given that GWAS catalog is manually curated from full-length articles while SNPcurator tool results are limited to the analysis of abstracts and titles only. Nevertheless, SNPcurator achieves a similar number of associations. It is important to note that SNPcurator results will almost certainly include false positives but as mentioned previously, reporting an association relies on a set of standards and rules that differ from one database to the other. SNPcurator results leave such evaluations to the user according to the information extracted. By observing the top 25 results when ordering associations in ascending order of their *P*-values, SNPcurator shows evidence for SNPs (rs8102683, rs465498 and rs12914385) and SNP (rs11127958) for the lung cancer and the obesity queries, respectively. Even though the GWAS catalog curation constraints might be the reason why these records are not listed in the catalog, we were still able to confirm these associations through other resources (6).

To further investigate the text-mining module performance, we compared the results from SNPcurator to associations derived from abstracts and titles only in the GWAS catalog without considering data from full text. The catalog cites a total of 37 articles for the obesity disease, only a subset of 22 articles were selected. A chosen article must include both the SNP id and the corresponding P-value in its abstract-text. SNPcurator was able to replicate 21 associations while 9 associations were missed or not properly extracted. For lung cancer search; 25 associations were matched from a total of 34 associations reported in GWAS catalog extracted from 28 articles. On average, SNPcurator was able to replicate around 70% of the associations.

### The performance of the information extraction component

Recently, SNPPhenA, a new corpus of SNPs and Phenotypes associations extracted from GWA studies was published online (http://nil.fdi.ucm.es/? q = node/639). The corpus was constructed by querying the GoPubMed (http://www.gopubmed.org/) with 20 popular SNPs fetched from SNPedia. The original 20 SNPs names were used as seeds for the abstract collection process that resulted in 360 abstracts with 875 distinct SNPs. The novelty of the SNPPhenA corpus relies in ranking the associations by classifying them into three classes: positive, negative and neutral. The associations were manually annotated by two experts in the genetic fields and in case of any contradictory results; the verdict of a third annotator was taken into consideration. Moreover, a confidence level of positive associations was manually extracted based on the strength of the linguistic correlation between SNPs and disease mentions in the abstract, they were categorized into weak, moderate and strong. More details on the annotation process and the corpus statistics can be found at (23).

To our knowledge, SNPPhenA is one the first datasets to include the degree of certainty and confidence of associations instead of only reporting binary relations that simply include association or no association between SNPs and disease. Despite the relevance of the dataset to our work, the corpus authors relied on linguistic information, negation, modality markers and neutral candidates to label associations. In our approach, we determine the significance of associations in terms of biomedical statistical tests and study size. It is worth mentioning that *P*-values were regarded as an extra factor by the annotators of SNPPhenA when identifying the confidence levels of reported associations.

To properly evaluate our model, only a subset of the corpus SNPPhenA_mod was used. All records of the original corpus with no p-values/OR values reported in the abstract text were excluded and not considered when building the new corpus. The modified corpus, SNPPhenA_mod, consists of 120 abstracts with 166 key sentences and a total of 331 SNP-Phenotype association candidates with 160 distinct SNP identifiers. The new corpus was constructed following the manual annotation of associations by a biological expert, itis available for download in XML format from the about page. We evaluated the performance of extracting both (<SNP, *P*-value>) pairs in terms of precision, recall and F-measure. The model achieved 81, 86 and 83%, respectively for each metric. What mostly affect the system sensitivity is the false negative (FN) cases that represents missed associations that were not detected by our system. Missed associations occurred due to failing to detect the *P*-value correctly or failing to link the correct *P*-value to the correct SNP. Failing to detect the correct *P*-value happens when values are reported in ranges instead of a single value. Another limitation of the system that it can only extract associations in the same sentence, i.e. both SNP and *P*-value are mentioned in the same sentence. It also assumes that one *P*-value applies to multiple SNPs if there is a single *P*-value mention with multiple SNPs mentioned in the sentence which in some cases results in False positives.On the other hand, failing to couple SNPs to their corresponding *P*-values contributes to a larger portion of (FN) and aslo to FP. Table 4 below illustrates some sample cases where SNPCurator failed to extract the correct associations

**Table 4.** Failed extraction cases

PubMed ID (PMID)	Evidence sentence	Reason
22914670	Two SNPs rs2656069 and rs10851906 in IREB2 were associated with COPD *P* = 0.045 and 0.032	Failure to detect the second *P*-value correctly led to missing the second association
23065249	The objective of this study was to investigate the coding region polymorphisms S19W (rs3135506) and G185C (rs2075291) and the promoter region polymorphism −1131T>C (rs662799) of the APOA5 gene as risk factors for ischemic stroke in Turkish population.…. 19W allele frequency was 0.090 in stroke patients and 0.062 in controls *P* = 0.191.	The authors used different terms for identifying both SNPs in question (S19, G185).
18820697	rs5770917, a SNP located between CPT1B and CHKB, was associated with narcolepsy in Japanese (rs5770917[C], OR = 1.79, combined P = 4.4 × 10(−7)) and other ancestry groups (OR = 1.40, *P* = 0.02).	The annotators matched SNP rs5770917C to the *P*-value while the system detected SNP rs5770917.
17383819	Significant association was detected at rs2254298 (*P* = 0.03) but not rs53576.	The system matched both SNP mentions to the *P*-value.

### External evaluation

Singhal et al. discuss in (24) the need to advance biomedical sciences through text mining by empowering the roles of stakeholders involved (researchers, publishers and experts). Domain experts can evaluate the mined results and provide requirements, comments and guidelines for future improvements. For that reason, we presented the SNPcurator website to a number of interested scientists to solicit their feedback through an online survey. All eight participants have doctoral degrees in genetics or biosciences and are currently involved in mutations and polymorphisms research. Their years of experience in the genetic field varied; one participant was a full professor, four lecturers and two assistant lecturers and a genetic engineering expert from industry. Three participants were affiliated with the genetics department in the Suez-Canal Faculty of Medicine, while two were affiliated with the genetics department in Alexandria faculty of Medicine, two with the Medical research institute in Alexandria and one with Molecular Medicine and Tissue Culture sector of the European Egyptian Pharmaceutical Industries.

**Figure 3.**
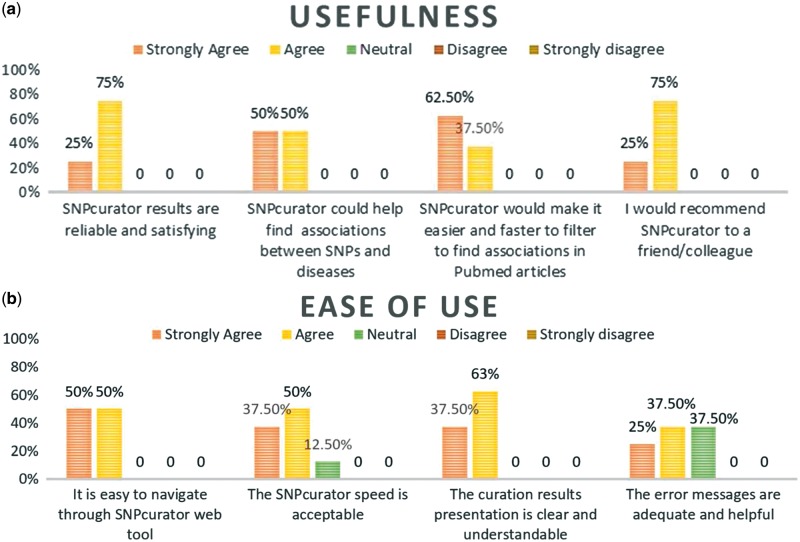
(a) Percentage of experts’ agreement with SNPcurator usefulness. **(b)** Percentage of experts’ agreement with SNPcurator ease of use.

The survey followed the original Davis technology acceptance model guidelines (25) by addressing its two main aspects; the perceived usefulness of the system and its ease of use. Moreover, it collected suggestions for improvements as well as their opinions on the context in which they envision using the tool. Participants were encouraged to try different diseases of their choice and evaluate the extracted results. The overall system performance was satisfactory to all of the users; Figure 3 demonstrates users’ responses to the questionnaire. Most of them agreed that SNPcurator would be useful for conducting a general preliminary research, studying SNPs variations among populations’ comparisons or highlighting related studies for further readings.

Some participants pointed out that the initial results were limited and some known associations were missing despite their mention in multiple citations. For that we increased the number of papers downloaded from PubMed through eUtils to 3000 instead of only 1000. This resulted in longer loading time but we asked them to repeat the experts search query and this time, the missing associations were present. They also suggested to extract more data from the literature to enrich the records such as minor allele frequency and phenotypic effect. However, this information is usually found in the full-text and not abstract and hence could not be implemented for now. Another suggestion was to enable users to search by SNPs not just disease, this would require us to implement a SNP-Trait association extraction module, which we intend to pursue in future work.

## Conclusion

In this article, we presented SNPcurator, a text-mining web-tool for automating the curation process of SNPs-trait information from GWASs. The system efficiently extracts associations and matches them with their statical significance parameters (*P*-value and OR) reported in abstracts; moreover it extracts population-related information such as the cohort sizes and ethnicity. To illustrate the tool’s usability, we compared the results of two sample queries as opposed to the manually curated database. The system was able to report a comparable number of associations and also recreate a number of existing associations found in GWAS catalog. The system was also able to identify new associations not found in the catalog that might be interesting for experts to further investigate. Furthermore, to evaluate its usefulness and ease of use in daily research practice, a group of experts used the tool to search for any disease of interest; their opinions were very encouraging and confirmed the potential for SNPcurator. The tool was proven scalable and robust by applying it on the whole PubMed repository, and increasing the Faded articles from 1000 to 3000 per query. A main limitation was the analysis of abstract text only, we believe more accurate data would be extracted from full-text articles.

We acknowledge the fact that text-mining systems would never step up to the level of sophistication of researchers when reading and analyzing an article and hence would never fully replace human curation. However, it is also almost impossible to keep up with the fast publication rates of scientific research today. The speed and satisfying results of SNPcurator may be very beneficial to accelerate that process.

## Future work

Results have shown a difference between the amount of associations reported in abstracts and those reported in the article text itself. In the future, we aim to expand the text-mining scope to full-length articles and also any extra data provided by authors to further improve the system’s performance. This would allow to extract more information regarding associations and also detailed cohort descriptions. Another potential add-on to the system would be a disease–SNP relation extraction module that allows an inverse query search by SNP id or even simply a set of PubMed articles ids. The system would also benefit from a general scoring scheme or a filter to rank the results not only based on the statistical findings but should incorporate as well the number of citations and the impact factors of the publications to indicate the credibility of the reported associations.

*Conflict of interest*. None declared.
