# Corrigendum to: the SNPcurator: literature mining of enriched SNP-disease associations

**DOI:** 10.1093/database/baab070

**Published:** 2021-11-24

**Authors:** Noha S Tawfik, Marco R Spruit

**Affiliations:** Computer Engineering Department, College of Engineering, Arab Academy for Science, Technology, and Maritime Transport (AAST), Abukir, Alexandria 1029, Egypt; Department of Information and Computing Sciences, Utrecht University, Princetonplein 5, Utrecht 3584 CC, The Netherlands; Department of Information and Computing Sciences, Utrecht University, Princetonplein 5, Utrecht 3584 CC, The Netherlands

Since this paper has published, the SNPcurator, which was previously hosted on http://snpcurator.science.uu.nl/, has moved. It is no longer accessible at http://snpcurator.science.uu.nl/ but can be found at http://tdslab.nl/snpcurator/.

